# Examining the diagnostic validity of the ISAAC asthma questionnaire among participants from Singapore and Malaysia

**DOI:** 10.5415/apallergy.0000000000000234

**Published:** 2025-12-02

**Authors:** Qi Yi Ambrose Wong, Dingyu Cen, Jun Jie Lim, Jia Yi Karen Wong, Kashaf Nadeem, Zheng Yang Ervin Yap, Jun Yan Ng, Yi Ying Eliza Lim, Yang Yie Sio, Yee How Say, Kavita Reginald, Fook Tim Chew

**Affiliations:** 1 Department of Biological Sciences, Faculty of Science, National University of Singapore, Singapore; 2 Department of Biological Sciences, School of Medical and Life Sciences, Sunway University, Petaling Jaya, Malaysia

**Keywords:** Allergy, asthma, point prevalence, sensitivity, specificity, questionnaire

## Abstract

**Background:**

Asthma is a chronic respiratory condition affecting 3,340 cases per 100,000 individuals globally. The significant global health burden of asthma necessitates the development and use of valid diagnostic tools for epidemiological purposes.

**Objective:**

This study evaluated the diagnostic accuracy of the International Study of Asthma and Allergies in Childhood (ISAAC) questionnaire among participants from the Singapore/Malaysia Cross-sequential Genetic Epidemiology Study (SMCGES).

**Methods:**

Investigators administered the ISAAC survey questionnaire to SMCGES participants. Symptoms related to asthma were compared against diagnosed asthma, which was characterized by a questionnaire-reported history of asthma and exhibiting a positive skin prick reaction to allergen extracts from 2 dust mite species. Diagnostic accuracy was assessed via sensitivity (Se), specificity (Sp), positive and negative predictive values, and Youden’s Index (*J*).

**Results:**

This study analyzed data from 4,028 participants from Malaysia and 11,473 participants from Singapore. Asthma symptom prevalence differed between Malaysia and Singapore, with ever wheeze being the most prevalent in both populations. Diagnosed asthma was more prevalent in Singapore (17.3%, 95% confidence interval [CI]: 16.6–18.1%) than in Malaysia (9.7%, 95% CI: 8.8–10.7%). Ever wheeze showed the best diagnostic performance for diagnosed asthma in Malaysia (Se = 0.548, Sp = 0.831, *J* = 0.379) and Singapore (Se = 0.679, Sp = 0.888, *J* = 0.566). Combining symptoms or asthma medication usage improved accuracy slightly, with the most valid combinations being ever wheeze or exercise-induced wheeze in Malaysia (Se = 0.557, Sp = 0.824, *J* = 0.381) and usage of bronchodilators, beta-agonists, inhaled steroids, or leukotriene modifiers in Singapore (Se = 0.771, Sp = 0.871, *J* = 0.642).

**Conclusion:**

The modified ISAAC questionnaire used by the SMCGES showed good Se and Sp for diagnosed asthma, especially when combining various symptom indicators or usages of different asthma medications. These findings substantiate the continued use of the ISAAC questionnaire as a valuable survey tool in epidemiological studies across diverse populations.

## 1. Introduction

### 1.1. Background

Asthma is an inflammatory airway condition that presents as wheezing, shortness of breath, chest tightness, and/or cough [1]. More than 260 million individuals worldwide have been estimated to be affected by asthma, while approximately 461,000 deaths have been attributed to asthma [2]. In 2021, there were approximately 3,340 asthma cases per 100,000 individuals around the globe [3]. To this day, asthma remains a major cause of morbidity across different age groups, contributing significantly to missed school and work, an increase in the utilization of healthcare services, and reduced quality of life [3, 4]. Due to the public health burden of asthma, there is an impetus for accurately identifying asthma cases for clinical management, epidemiological research, and public health planning [5, 6]. Standardized tools, notably the International Study of Asthma and Allergies in Childhood (ISAAC) survey for investigating and monitoring allergic diseases, including asthma, have been developed for large-scale epidemiological studies across different populations [7]. While initially targeted at children, the ISAAC questionnaire has been adapted for broader use; ISAAC-based studies have now focused on older adult populations and diverse language settings [8–10]. A corollary to the usage of ISAAC in varying cultural settings is a likely change in its diagnostic validity indices (ie, sensitivity (Se), specificity (Sp), positive predictive value [PPV], and negative predictive value [NPV]). Currently, the Singapore/Malaysia Cross-sequential Genetic Epidemiology Study (SMCGES) is investigating allergic diseases in 2 distinct populations from Malaysia and Singapore. Since the validity of the ISAAC questionnaire varies across populations, the current analysis evaluated its validity among SMCGES participants from Malaysia and Singapore.

## 2. Methods

### 2.1. Ethics committee approval

The National University of Singapore Institutional Review Board (NUS-IRB) reviewed and approved this study (NUS-IRB address: Centre for Life Sciences, 28 Medical Drive, Singapore 117456; NUS-IRB Chairperson: Professor Paul Tambyah). The codes and dates of the relevant IRB approvals were NUS-07-023 (2007), NUS-09-256 (2009), NUS-10-445 (2010), NUS-13-075 (2013), NUS-14-150 (2014), and NUS-18-036 (2018). The current study complied with the Declaration of Helsinki. The research protocol also followed Good Clinical Practice guidelines and Singapore regulations. Every study participant was thoroughly apprized of the study details, and participation in the study was only permitted after the participant gave informed written consent.

### 2.2. Participant recruitment

The SMCGES is a cross-sequential epidemiological research project currently taking place in 3 universities: (i) the National University of Singapore (NUS), Singapore; (ii) Universiti Tunku Abdul Rahman, Malaysia; and (iii) Sunway University, Malaysia. Individuals from across the university campuses were invited to participate in the SMCGES via posters and email advertisements. These subjects were screened against predetermined inclusion and exclusion criteria. Subjects fulfilled the inclusion criteria if they were: (i) at least 18 years of age, (ii) random individuals unrelated to any other subject already enrolled in the SMCGES, (iii) willing to respond to all questions from our study questionnaire, and (iv) had no needle phobia. Our exclusion criteria were: participant had either (i) had a relation to any subject who had enrolled in the SMCGES, or (ii) had a known genetic disease or syndrome, or (iii) had previously participated in the SMCGES, or (iv) had a needle phobia, or (v) had taken antihistamines within 3 days before the skin prick test (SPT), or (vi) were unwilling to provide epidemiological data. Participants who ceased their antihistamine consumption returned at a later date, at least 2 days after cessation, to complete the study.

### 2.3. Data collection

Participants were administered a modified ISAAC Phase Three Survey [11]. Per the established and standardized protocol detailed by the ISAAC Steering Committee, epidemiological and symptomological histories were collected from the study subjects [8]. Subject histories of previous asthma medication prescriptions, including bronchodilators, short-acting and long-acting beta-agonists, inhaled steroids, and leukotriene modifiers, were also obtained. Sensitization to house dust mite allergens was assessed using a standardized skin prick testing protocol reported in previous SMCGES publications [12–14].

Per ISAAC protocol, several asthma-related outcomes were identified: ever wheeze was classified when the participant had exhibited wheezing or whistling in the chest at any time in the past; current wheeze was classified when the participant had had wheezing or whistling in the chest in the last 12 months; sleep disturbances due to wheeze was classified when the participant indicated experiencing sleep disturbances due to wheezing in the past 12 months; speech disruptions due to wheeze was classified when the participant had ever experienced wheezing severe enough to limit their speech to only 1 or 2 words at a time between breaths; exercise-induced wheeze was classified when the participant’s chest had ever sounded wheezy during or after exercise in the past 12 months; and dry nocturnal cough was classified when the participant had ever experienced a dry cough at night, apart from a cough associated with a cold or chest infection [15]. Subjects who further confirmed and responded affirmatively to the question “Have you ever had asthma?” were considered to have a history of asthma. Diagnosed asthma cases comprised individuals with a history of asthma who exhibited a positive SPT reaction to house dust mite extract.

### 2.4. Statistical analysis

The SMCGES database was analyzed using R version 4.4.0 (R Foundation for Statistical Computing, Vienna, Austria [16]. Prevalences and accompanying confidence intervals (CIs) were calculated for the asthma-related outcomes described in the section above (Data collection) [17]. Statistical comparisons of prevalences were performed using Pearson’s chi-squared tests. Asthma symptom questions were assessed against diagnosed asthma using 5 measures of diagnostic validity. These include the Se, the proportion of affirmative responses among those with diagnosed asthma; Sp, the proportion of negative responses among those without diagnosed asthma; PPV, the probability that diagnosed asthma in subjects responded affirmatively; and NPV, which represents the proportion of healthy individuals without diagnosed asthma out of those who responded negatively [18]. The Youden’s Index was obtained by deducting 1 from the sum of Se and Sp [18].

## 3. Results

The SMCGES sample comprises 4,028 subjects aged 18 to 77 years from Malaysia and 11,473 individuals aged 18 to 81 years from Singapore (hereafter referred to as Malaysian participants and Singaporean participants, respectively). The interquartile ranges for the age at participation were 19 to 22 years in Malaysia and 20 to 23 years in Singapore. Singaporean participants had a greater mean age than Malaysian participants (mean age and standard deviation of the Singapore subset vs that of Malaysia: 22.8 ± 5.7 vs 21.1 ± 4.6; *P* < 0.001). Malaysian participants were more likely to be female than Singaporean participants (proportion of females: 65.0% vs 58.1%; *P* < 0.001).

Among asthma symptoms in Malaysian participants, the prevalence of ever wheeze was the highest at 20.8% (95% CI: 19.5–22.2), while exercise-induced wheeze was the lowest at 11.6% (95% CI: 10.6–12.7). Similarly, ever wheeze was the most prevalent asthma symptom among Singapore participants at 18.9% (95% CI: 18.2–19.6), and exercise-induced wheeze was the least frequent asthma symptom at 4.0% (95% CI: 3.6–4.4). Additionally, ever wheeze, current wheeze, exercise-induced wheeze, and dry nocturnal cough were all significantly more prevalent among Malaysian participants than Singaporean participants (see Table 1).

**Table 1.** A summary of prevalences for asthma symptoms

	Prevalence (95% CI) (%)		*P*-value*
	Malaysia (n = 4,028)	Singapore (n = 11,473)	
Ever wheezing	20.8 (19.5–22.2)	18.9 (18.2–19.6)	0.009
Current wheeze	15.8 (14.7–17.1)	9.2 (8.6–9.8)	<0.001
Sleep disturbances due to wheeze	2.3 (1.9–2.9)	2.6 (2.2–2.9)	0.50
Speech disruptions due to wheeze	7.3 (6.5–8.2)	6.6 (6.1–7.2)	0.17
Exercise-induced wheeze	11.6 (10.6–12.7)	4.0 (3.6–4.4)	<0.001
Dry nocturnal cough	13.2 (12.2–14.4)	11.4 (10.8–12.1)	0.005

CI, confidence interval.

* Pearson’s chi-squared test *P*-values for the comparison of prevalences between Malaysian subjects and Singaporean subjects.

Diagnosed asthma prevalence was 9.7% (95% CI: 8.8–10.7) and 17.3% (95% CI: 16.6–18.1) in Malaysia and Singapore, respectively. Amongst ISAAC asthma symptoms, ever wheeze showed the best diagnostic performance for diagnosed asthma in Malaysia (Se = 0.548, Sp = 0.831, *J* = 0.379; Table 2) and Singapore (Se = 0.679, Sp = 0.888, *J* = 0.566; Table 2). Different alternative criteria for asthma using symptomological information obtained through the ISAAC questionnaire were also tested – in Malaysia, the combinations of ever wheeze or exercise-induced wheeze (Se = 0.557, Sp = 0.824, *J* = 0.381) and ever wheeze or asthma medication prescription (Se = 0.827, Sp = 0.546, *J* = 0.373) showed the best diagnostic performance for diagnosed asthma (see Table 3). In Singapore, combinations of any asthma medication prescription (Se = 0.771, Sp = 0.871, *J* = 0.642) and ever wheeze or exercise-induced wheeze (Se = 0.687, Sp = 0.871, *J* = 0.557) were the most valid tests for diagnosed asthma (see Table 3).

**Table 2.** Sensitivity, specificity, positive predictive value, negative predictive value, and Youden’s Index for asthma symptom questions, using diagnosed asthma as a reference

	Malaysia					Singapore				
	Se	Sp	PPV	NPV	*J*	Se	Sp	PPV	NPV	*J*
Ever wheezing	0.548	0.831	0.261	0.944	0.379	0.679	0.888	0.558	0.930	0.566
Current wheeze	0.302	0.858	0.189	0.918	0.160	0.215	0.936	0.448	0.832	0.151
Sleep disturbances due to wheeze	0.341	0.858	0.375	0.839	0.199	0.310	0.819	0.532	0.642	0.129
Speech disruptions due to wheeze	0.151	0.945	0.394	0.824	0.096	0.093	0.951	0.547	0.624	0.044
Exercise-induced wheeze	0.211	0.895	0.184	0.910	0.106	0.096	0.973	0.460	0.819	0.069
Dry nocturnal cough	0.229	0.879	0.176	0.910	0.108	0.178	0.901	0.299	0.822	0.079

J, Youden’s Index; NPV, negative predictive value; PPV, positive predictive value; Se, sensitivity; Sp, specificity.

**Table 3.** Sensitivity, specificity, positive predictive value, negative predictive value, and Youden’s Index for operational definitions of asthma based on different combinations of questions and/or medication prescriptions, using diagnosed asthma as the reference

	Malaysia					Singapore				
	Se	Sp	PPV	NPV	*J*	Se	Sp	PPV	NPV	*J*
Any asthma medication prescription*	0.233	0.955	0.369	0.916	0.188	0.771	0.871	0.629	0.931	0.642
Ever wheeze or exercise-induced wheeze	0.557	0.824	0.261	0.943	0.381	0.687	0.871	0.558	0.921	0.557
Ever wheeze or dry nocturnal cough	0.612	0.752	0.217	0.945	0.364	0.708	0.802	0.459	0.920	0.510
Ever wheeze, exercise-induced wheeze, or dry nocturnal cough	0.615	0.751	0.217	0.946	0.366	0.708	0.801	0.459	0.920	0.509
Ever wheeze or asthma medication prescription*	0.827	0.546	0.256	0.943	0.373	0.961	0.528	0.544	0.958	0.489
Ever wheeze, exercise-induced wheeze, or asthma medication prescription*	0.833	0.523	0.256	0.941	0.356	0.961	0.524	0.544	0.957	0.485
Ever wheeze, dry nocturnal cough, or asthma medication prescription*	0.862	0.415	0.214	0.942	0.277	0.964	0.411	0.455	0.957	0.375
Ever wheeze, exercise-induced wheeze, dry nocturnal cough, or asthma medication prescription*	0.862	0.414	0.214	0.942	0.276	0.964	0.411	0.455	0.957	0.375
Exercise-induced wheeze or dry nocturnal cough	0.349	0.807	0.169	0.917	0.156	0.222	0.887	0.318	0.827	0.108
Exercise-induced wheeze, dry nocturnal cough, or asthma medication prescription*	0.717	0.496	0.175	0.922	0.214	0.878	0.540	0.384	0.931	0.417

J, Youden’s Index; NPV, negative predictive value; PPV, positive predictive value; Se, sensitivity; Sp, specificity.

* Asthma medications refer to bronchodilators, short-acting beta-agonists, long-acting beta-agonists, inhaled steroids, and leukotriene modifiers.

## 4. Discussion

### 4.1. Prevalence rates of symptoms related to asthma and diagnosed asthma

The current analysis evaluated asthma prevalence in Malaysia and Singapore, respectively. Among ISAAC asthma symptoms, ever wheeze was the most prevalent at 20.8% in Malaysia and 18.9% in Singapore. Current wheeze prevalence was lower than ever wheeze for both Malaysia (15.8% vs 20.8%) and Singapore (9.2% vs 18.9%).

Although the higher rates of asthma symptoms in Malaysia compared to Singapore set grounds for the assumption that diagnosed asthma prevalence would be higher in Malaysia, our findings showed a contrasting trend where diagnosed asthma was significantly less prevalent in Malaysia than in Singapore (9.7% vs 17.3%). One plausible explanation for this discrepancy lies in the differences in access to healthcare and diagnostic practices between the 2 countries. The Singaporean Ministry of Health has recognized the increasing burden of asthma and implemented the Singapore National Asthma Program (SNAP) to support primary care practices in the diagnosis, tracking, and management of asthma in the population [19, 20]. Education campaigns conducted through SNAP have likely further improved asthma awareness and recognition [21]. Conversely, asthma diagnosis in Malaysia is hindered by barriers to healthcare utilization, especially in rural areas, and by challenges to implementing asthma guidelines among healthcare providers [22, 23]. Cultural differences in healthcare-seeking behavior, further compounded by socioeconomic status, may also influence the likelihood of receiving an asthma diagnosis. However, this hypothesis requires confirmation by studies of the psychosocial determinants of undiagnosed asthma cases.

The current study joins the ranks of epidemiological studies worldwide utilizing the ISAAC asthma questionnaire [24]. In Asia, ever wheeze, current wheeze, and ever asthma in separate populations have also been studied using the ISAAC questionnaire [25–27]. Results from these studies showed that ever wheeze and current wheeze in Singapore have been consistently higher than in the surrounding Asia region [28]. Correspondingly, ever asthma in Singapore has also been among the highest in Asia [28]. In contrast to the reported trend, our results show that ever wheeze and current wheeze in Malaysia may be on the rise, with the accompanying prevalence rates surpassing those of Singapore. While ever asthma prevalence, which may encapsulate individuals who are aware of their asthma status, remains higher in Singapore than in Malaysia, that is projected to change based on the current prevalence rates of ever wheeze and current wheeze in Malaysia.

### 4.2. Diagnostic performance of the ISAAC questionnaire as compared to previous studies

Due to the widespread use of the ISAAC questionnaire and its entailing translation into various local languages, several reports assessing diagnostic performance have been published in the scientific literature [29]. However, few reports exist for the diagnostic performance of the English version [30, 31]. Wheezing in the past 12 months is in close agreement with diagnosed asthma among children (Se = 0.85, Sp = 0.81, PPV = 0.61, NPV = 0.94, *J* = 0.66) and adults (Se = 0.80, Sp = 0.97, PPV = 0.89, NPV = 0.94, *J* = 0.76) [30]. In contrast, a separate study using the methacholine challenge as the reference test found that none of the ISAAC asthma questions were sufficiently sensitive or specific to be used in establishing an asthma diagnosis (ever wheezing: Se = 73%, Sp = 11%, PPV = 50%, NPV = 25%; wheezing in the past 12 months: Se = 75%, Sp = 13%, PPV = 46%, NPV = 33%; ever asthma: Se = 36%, Sp = 78%, PPV = 67%, NPV = 50%) [31].

Similarly, non-English versions of the ISAAC questionnaire have been subjected to validity studies. A 2011 report on 5-year-olds using a Finnish ISAAC questionnaire showed acceptable validity of the questions on any wheezing symptoms in the past 12 months (Se = 0.88, Sp = 0.75, PPV = 0.13, NPV = 0.99, *J* = 0.63) and ever asthma (Se = 0.98, Sp = 0.97, PPV = 0.57, NPV = 1.00, *J* = 0.95) against a doctor-diagnosed asthma [32]. In this study, the questionnaire-reported ever doctor-diagnosed asthma was comparable to that of ever asthma in terms of validity against a doctor’s diagnosis of asthma (Se = 0.98, Sp = 0.97, PPV = 0.58, NPV = 1.00, *J* = 0.96) [32]. A combination of wheeze symptoms or usage of any medications prescribed for asthma, both during the last 12 months, plus a history of doctor-diagnosed asthma further improved the validity in establishing an asthma diagnosis (Se = 0.98, Sp = 0.98, PPV = 0.64, NPV = 1.00, *J* = 0.96) [32]. Another study of Brazilian children aged 12 to 19 featured a Portuguese version of the ISAAC questionnaire [33]. Therein, using physician-diagnosed asthma as the reference test, ever wheezing was the most sensitive and specific (Se = 80.6%, Sp = 74.9%, PPV = 56.4%, NPV = 9.4%, *J* = 0.55); however, asthma symptoms in the past 12 months all exhibited low Se, with the best question being wheezing in the past 12 months (Se = 44.2%, Sp = 90.0%, PPV = 64.0%, NPV = 20.0%, *J* = 0.34) [33]. Finally, a validation of the Spanish ISAAC questionnaire showed that ever wheezing (Se = 93.3%, Sp = 89.9%, PPV = 79.7%, NPV = 96.9%, *J* = 0.83) and ever asthma (Se = 96.2%, Sp = 96.0%, PPV = 90.9%, NPV = 98.3%, *J* = 0.92) had the highest validity for a clinically diagnosed asthma [34]. Wheezing in the past 12 months had a lower Se for diagnosed asthma (Se = 65.3%, Sp = 91.3%, PPV = 91.4%, NPV = 64.9%, *J* = 0.57) [34]. Overall, there has been a consensus that the ISAAC questionnaire in its English and non-English iterations is suitable for preliminary asthma diagnoses, especially in the context of epidemiological studies.

The current results showed moderate diagnostic performance of the ISAAC questionnaire for diagnosed asthma in Malaysia and Singapore. Presently, the validity metrics for some questions are broadly comparable to those of the preexisting literature for English and non-English versions of the ISAAC questionnaire. Specifically, ever wheeze, the best-performing question in Singapore (Se = 0.679, Sp = 0.888, *J* = 0.566) was comparable to the validation results of Lukrafka et al. [33] (Se = 80.6%, Sp = 74.9%, *J* = 0.55). Conversely, original ISAAC validation results for current wheeze were not replicated in the SMCGES cohort (ie, the Se and Sp of current wheeze in Malaysia and Singapore were lower than those in Jenkins et al. [30]). Alternative combinatory criteria that included different symptoms were also tested based on findings of the European Community Respiratory Health Survey (ECRHS), which showed that combinations of questions performed better than single-symptom questions at detecting clinician-diagnosed current asthma [35]. Therein, combining ECRHS questions for wheeze, shortness of breath, and participants’ self-reports of current asthma (Se = 82.9%, Sp = 86.7%, *J* = 0.70) resulted in better diagnostic performance than the best single-symptom question (wheeze in the past 12 months: Se = 68.6%, Sp = 91.2%, *J* = 0.60) or ever asthma (Se = 67.6%, Sp = 97.5%, *J* = 0.65) [35]. Similarly, combinatory questions from the present analysis improved diagnostic performance, with the best combinations being ever wheeze or exercise-induced wheeze in Malaysia (Se = 0.557, Sp = 0.824, *J* = 0.381) and any asthma medication prescription in Singapore (Se = 0.771, Sp = 0.871, *J* = 0.642).

The current PPVs across symptom questions (Malaysia: 0.176–0.394; Singapore: 0.299–0.558) and combination questions (Malaysia: 0.169–0.369; Singapore: 0.318–0.629) replicate findings of previous validation studies, where PPVs tend to be low in populations with relatively low disease prevalence [32, 33]. In contrast, the NPV was at least 0.90 across most tests in Malaysia and Singapore. This observation reiterates a key limitation in utilizing the questionnaire for asthma diagnosis in individuals: the questionnaire retains its utility in identifying nonasthmatic individuals, but its lack of precision in diagnosing diagnosed asthma emphasizes the importance of confirming the asthma diagnosis with a physician’s examination, objective asthma tests, or both. Hence, our results support the consensus that the ISAAC questionnaire remains more appropriate for population-level screening or epidemiological studies rather than definitive clinical diagnoses.

### 4.3. Diagnosed asthma as a reference test for validation in our study

Until recently, the diagnosis of asthma has been complicated by the absence of standardized diagnostic criteria [6, 36]. In efforts to standardize the diagnosis of asthma, since 1989, organizations such as the American Thoracic Society/European Respiratory Society, British Thoracic Society/Scottish Intercollegiate Guidelines Network, and Global Initiative for Asthma (GINA) have each issued separate guidelines [36, 37]. For example, the diagnostic guideline published by GINA requires both a history of wheeze, shortness of breath, chest tightness, and/or cough, and a confirmed expiratory airflow limitation for a definitive diagnosis of asthma [1]. Assessing these guidelines using a reference standard of asthma diagnosed by a panel of asthma specialists showed that while these guidelines share overlapping criteria, their Se and Sp for asthma varied notably [38]. Moreover, the concordance between different guidelines was only moderate [38]. Despite the contradictions between guidelines, there has been a clear shift towards objective tests for asthma with a recommended consideration of the symptomological history.

The lack of objective asthma measures poses a limitation in the current study. Objective asthma tests, such as spirometry tests or methacholine challenge tests (MCTs), ostensibly offer better diagnostic accuracy and strengthen the validity of the diagnosis. However, these tests require greater effort, human resources, and time investment, all of which are in limited supply, especially in the context of our large-scale study. Furthermore, these tests have not reliably shown utility in definitively diagnosing asthma. Indeed, the spirometry test showed a low Se (23%) when assessed against a reference diagnosis using a positive result from the MCT and the presence of asthma symptoms [39]. Furthermore, there are reports of the MCT being an unsatisfactory marker of asthma [40]. In consideration of the limitations of objective asthma testing and their associated resource burden, the SMCGES utilizes the SPT as an alternative objective measure. The SPT tests for atopy, to which a close association with asthma and asthma-related bronchial hyperresponsiveness has been reported in the scientific literature [41, 42]. The extracts used in the SPT were from *Blomia tropicalis* and *Dermatophagoides pteronyssinus*, both of which are dust mite species occurring frequently in the SMCGES setting and to which there is a high rate of sensitization in the local atopic populations [43, 44]. Although the allergen panel was limited to only 2 dust mites, forgoing other allergens such as cockroach, cat fur, dog fur, molds, grass pollens, or Acacia, this was sufficient for the identification of atopy since allergy in the tropical urban environment of Malaysia and Singapore have been found to be primarily driven by mono-specific IgE sensitization against house dust mites [45]. The combination of objective results from the SPT with data from the ISAAC questionnaire thus strengthens the screening of asthma in the context of epidemiological research.

## 5. Limitations and conclusion

This analysis draws on the extensive samples collected from Malaysia and Singapore as part of the large-scale SMCGES project. The results from the present study thus carry statistical power enhanced by the large sample size. Additionally, by consistently applying the same ISAAC protocol, we could assess the validity of the ISAAC questionnaire in Singapore and Malaysia with minimal confounding from varying practices between research teams. This study was limited by the lack of objective asthma measures, such as the spirometry test or MCT, which offer better diagnostic accuracy and strengthen the validity of the diagnosis. Moreover, the allergen panel used to construct the reference standard in this analysis was restricted to *B. tropicalis* and *D. pteronyssinus*. Nonetheless, we have comprehensively assessed the diagnostic utility of the ISAAC questionnaire for asthma and how different operational definitions based on combinations of the questions may alter the diagnostic validity in identifying asthma cases. These results represent a current assessment of the ISAAC asthma questionnaire’s performance in Malaysia and Singapore. Metrics of the diagnostic performance were comparable to those of previous validation studies, and the questionnaire effectively ruled out asthma among subjects. However, asthma identified solely through the questionnaire data cannot constitute a definitive diagnosis, and a “true” diagnosis of asthma should comprise a physician’s examination, objective asthma tests, or both. Notwithstanding, the current findings substantiate the continued use of the ISAAC questionnaire as a valuable screening tool in epidemiological studies across diverse populations.

## Acknowledgements

We are deeply appreciative of all participants involved in the SMCGES. Our gratitude extends to the research members involved in this study, including all undergraduate and master’s students from the National University of Singapore Functional Genomics Lab, and Felicia Ooi Ze En, Wong Yi Ru, and Smyrna Moti Rawanan Shah from Sunway University, for their time and effort invested in this research project.

FTC received funding from the National University of Singapore, Singapore Ministry of Education Academic Research Fund, Singapore Biomedical Research Council, Singapore Immunology Network, Singapore National Medical Research Council, Singapore National Research Foundation, Singapore Food Agency, Singapore Economic Development Board, and the Singapore Agency for Science Technology and Research. This research is supported by the National Research Foundation Singapore under its Open Fund-Large Collaborative Grant (MOH-001636) (A-8002641-00-00) and administered by the Singapore Ministry of Health’s National Medical Research Council. KR has received funding from the T20 Research Collaboration Grant Scheme from Sunway University. These organizations were not involved in any aspect of the work reported in this manuscript.

## Conflicts of interest

FTC reports grants from the National University of Singapore, Singapore Ministry of Education Academic Research Fund, Singapore Immunology Network, Singapore National Medical Research Council, Singapore Biomedical Research Council, Singapore National Research Foundation, Singapore Food Agency, Singapore Economic Development Board, and the Singapore Agency for Science Technology and Research. These grants were in effect during this study. FTC also reports receiving consulting fees from Sime Darby Technology Centre, First Resources Ltd, Genting Plantation, Olam International, Musim Mas, and Syngenta Crop Protection. The remaining authors declare no conflicts of interest.

## Author contributions

Fook Tim Chew conceived and supervised the current research study. Qi Yi Ambrose Wong conducted the literature review, analyzed and interpreted the data, and wrote the manuscript. Qi Yi Ambrose Wong, Dingyu Cen, Jun Jie Lim, Jia Yi Karen Wong, Kashaf Nadeem, Zheng Yang Ervin Yap, Jun Yan Ng, Yi Ying Eliza, Yang Yie Sio, Yee How Say, and Kavita Reginald assisted in recruiting study participants and data collation. All authors read and approved of the final manuscript.
